# Pneumoproteins are associated with pulmonary function in HIV-infected persons

**DOI:** 10.1371/journal.pone.0223263

**Published:** 2019-10-01

**Authors:** Diane Jeon, Emily G. Chang, Maggie McGing, Marlena Hartman-Filson, Mathew Sommers, Eula Lewis, John R. Balmes, Daniela Moisi, Michael M. Lederman, Kristine A. Madsen, Prescott G. Woodruff, Peter W. Hunt, Laurence Huang, for the Inflammation, Aging, Microbes and Obstructive Lung Disease (I AM OLD) Study

**Affiliations:** 1 University of California Berkeley-University of California San Francisco Joint Medical Program, Berkeley, California, United States of America; 2 HIV, ID and Global Medicine Division, Department of Medicine, Zuckerberg San Francisco General Hospital and Trauma Center, University of California San Francisco, San Francisco, California, United States of America; 3 Department of Anesthesia and Perioperative Care, Zuckerberg San Francisco General Hospital and Trauma Center, University of California San Francisco, San Francisco, California, United States of America; 4 School of Public Health, University of California Berkeley, Berkeley, California, United States of America; 5 Division of Pulmonary and Critical Care Medicine, Department of Medicine, Zuckerberg San Francisco General Hospital and Trauma Center, University of California San Francisco, San Francisco, California, United States of America; 6 Department of Medicine, Case Western Reserve University, Cleveland, Ohio, United States of America; 7 Division of Pulmonary and Critical Care Medicine, Department of Medicine, University of California San Francisco, San Francisco, California, United States of America; 8 Division of Experimental Medicine, Department of Medicine, University of California San Francisco, San Francisco, California, United States of America; Northwestern University, UNITED STATES

## Abstract

**Background:**

COPD is a common HIV comorbidity, and HIV-infected individuals have a higher incidence and earlier onset of COPD compared to HIV-uninfected individuals. While the pathogenesis of HIV-associated COPD is largely unknown, chronic inflammation may contribute. Four pneumoproteins known to be markers of lung injury and inflammation have been associated with COPD in HIV-uninfected individuals: PARC/CCL-18, SP-D, CC-16, and sRAGE.

**Objective:**

To determine whether these pneumoproteins are also associated with pulmonary function and COPD Assessment Test (CAT) scores in HIV-infected individuals.

**Methods:**

Associations between plasma pneumoprotein levels and pulmonary function were determined in a cross-sectional study of otherwise healthy HIV-infected individuals enrolled between September 2016 and June 2017. Covariates included HIV-associated (antiretroviral therapy, CD4 count, and viral load) and COPD-associated (smoking and BMI) covariates.

**Results:**

Among 65 participants, 78.5% were male, 50.8% had undetectable viral load, and 76.9% were ever-smokers. Mean post-bronchodilator FEV_1_/FVC was 0.71, and mean DLco%predicted was 61%. Higher PARC/CCL-18 was associated with lower DLco%predicted and higher CAT score. Higher CC-16 was associated with lower DLco%predicted and lower FVC%predicted.

**Conclusions:**

This exploratory analysis is the first to characterize associations between these four pneumoproteins and pulmonary function in an HIV-infected cohort. Our findings suggest the pathogenesis of HIV-associated COPD may differ from that of non-HIV-associated COPD due to HIV-specific inflammatory changes affecting DLco. PARC/CCL-18 is associated with structural and functional pulmonary abnormalities and may be an important COPD biomarker candidate in HIV infection. Our study is a preliminary step toward finding clinically relevant COPD biomarkers in high-risk populations.

## Introduction

Chronic obstructive pulmonary disease (COPD) causes significant morbidity and mortality globally.[1–3] HIV infection may be an independent risk factor for COPD.[4–12] Studies of HIV-infected persons in the U.S. have reported estimates for COPD prevalence ranging between 7% and 21%[7,13,14] compared to between 4.2% and 6.4% in the general population.[15,16] Even when controlling for major COPD risk factors such as smoking status, HIV-infected individuals tend to develop COPD on average 10 years earlier than HIV-uninfected individuals.[11,12] Thus, HIV-infected individuals are an important high-risk population in which to study the pathogenesis of COPD.

Despite substantial epidemiological evidence linking HIV and COPD, the mechanisms underlying HIV-associated COPD (HIV-COPD) and whether these mechanisms differ from those in HIV-uninfected persons are not fully understood.[17,18] HIV is known to promote systemic immune activation and inflammation leading to immunosenescence or immune dysregulation that increase the risk of end-organ diseases.[17–23] Proposed mechanisms specific for HIV-associated COPD include endothelial dysfunction, monocyte activation,[24] and T-cell activation.[10] Understanding the role of systemic inflammation in HIV-associated COPD pathogenesis may yield insights about other HIV-associated pulmonary abnormalities including reductions in the diffusing capacity for carbon monoxide, DLco, as an isolated decrease in DLco is the most frequent pulmonary function abnormality observed in HIV-infected populations.[4,10,20,24]

Spirometry measurements, specifically the ratio of forced expiratory volume in 1 second to forced vital capacity (FEV_1_/FVC) measured after bronchodilator administration and FEV_1_ as a percentage of the predicted value (FEV_1_%predicted) are used to diagnose and stage the severity of COPD, respectively.[25] However, due to the heterogeneity of COPD pathogenesis, the FEV_1_/FVC is not always an accurate predictor of progression of COPD and other associated clinical outcomes such as symptoms, COPD exacerbations, or mortality.[26,27] Thus, recent COPD studies have focused on identifying biomarkers that are associated with or predictive of key clinical endpoints.[28–30]

The most promising COPD biomarker candidates include pneumoproteins associated with systemic inflammation. Pneumoproteins are produced predominantly in the lung but are present in the systemic circulation. Four pneumoproteins of interest for COPD are pulmonary and activation-regulated chemokine (PARC/CCL-18), surfactant protein-D (SP-D), club cell secretory protein-16 (CC-16), and soluble receptor for advanced glycation end-products (sRAGE). PARC/CCL-18 and SP-D are pro-inflammatory proteins, and CC-16 and sRAGE are anti-inflammatory proteins. All of these pneumoproteins are markers of lung injury and inflammation that have been independently associated with COPD-related outcomes in HIV-uninfected populations including severity of emphysema (sRAGE),[31] accelerated rate of FEV1 decline (CC-16),[32,33] increased risk of COPD exacerbations (SP-D),[34] and cardiovascular hospitalization and mortality (PARC/CCL-18).[35]

None of these pneumoproteins, however, has been studied for its association with COPD in HIV-infected individuals. Thus, this cross-sectional analysis of an existing HIV-infected COPD cohort investigated whether plasma levels of these four pneumoproteins are associated with pulmonary function and respiratory symptoms. Since HIV is known to increase chronic systemic inflammation, we hypothesized that HIV may affect the relationship between these markers of lung inflammation and pulmonary function outcomes.

## Methods

### Study design, participants, and study protocol

This study is a cross-sectional analysis of the San Francisco arm of the Inflammation, Aging, Microbes, and Obstructive Lung Diseases (I AM OLD) study, an ongoing study of COPD in HIV-infected individuals. Participants gave written informed consent, and the study protocol was approved by the University of California San Francisco Institutional Review Board and the University of California Berkeley Institutional Review Board. I AM OLD participants are recruited as inpatients when hospitalized for confirmed pneumonia or as outpatients in the HIV/AIDS clinic at Zuckerberg San Francisco General Hospital (ZSFG) and followed longitudinally as one of the study aims is examining whether pneumonia is a risk factor for COPD in HIV. Study visits occurred at ZSFG between September 2016 and June 2017. Inclusion criteria included HIV-infected status and 18 years of age or older. Exclusion criteria included the presence of any contra-indications for pulmonary function tests (PFTs), pregnancy, or breastfeeding.

For participants enrolled during a hospitalization for pneumonia, study visits for PFTs occurred at least 3 months after the end of pneumonia treatment and when they were without acute or worsening chronic respiratory symptoms. At each visit, participants underwent clinical questionnaires, venous blood draws for pneumoprotein measurements, and PFTs.

### Clinical questionnaires

Participants were interviewed by trained staff using a standardized questionnaire. Clinical data included age, sex, race/ethnicity, history of ever cigarette smoking, cumulative pack-years of cigarette smoking, history of injection drug use (IDU), history of bacterial pneumonia or *Pneumocystis jirovecii* pneumonia (PCP), and self-reported ART adherence in the past week (yes/no response to any antiretroviral medication use in the past week). The COPD Assessment Test (CAT), a validated questionnaire that measures a patient’s self-assessment of the impact of COPD on their quality of life, was also administered.[36] CD4 count and HIV viral load as well as body mass index (BMI) were measured at the time of pulmonary function testing.

### Blood draws and ELISA assays

Blood was collected in an 8 mL EDTA tube. Specimens were placed on ice and centrifuged within an hour after collection. Purified plasma aliquots were then stored at -80°C, shipped to Case Western Reserve University, and assayed after a single thaw. Levels of pneumoproteins were measured in purified plasma using commercial ELISA kits (R&D, Minneapolis, Minnesota, USA). Replicates were performed in duplicate, and average values were used in analyses.

### Pulmonary function tests

Participants were without acute or worsening chronic respiratory symptoms at the time of PFTs. PFTs consisted of pre- and post-bronchodilator spirometry and measurement of diffusing capacity for carbon monoxide (DLco). We did not measure total lung capacity or residual volume. Spirometry was performed by trained respiratory technicians, before and after administering albuterol 360 μg by inhalation from metered dose inhaler. Spirometry and DLco measurements were performed according to American Thoracic Society/European Respiratory Society (ATS/ERS) guidelines.[37,38] Spirometry reference values were determined from the third National Health and Nutrition Examination Survey equations and are based on age, sex, height, and ethnic background.[39] DLco reference values were determined from Crapo et al. 1981 and based on age, sex, and height.[40] The DLco reference values were also adjusted for hemoglobin and carboxyhemoglobin that were measured at the time of pulmonary function testing. Spirometry results were overread by a trained respiratory therapist and included only if they met ATS/ERS criteria for acceptability and reproducibility.

### Statistical analysis

Analyses were conducted in Stata version 14.0 (StataCorp; College Station, Texas, USA). Pneumoprotein levels were log-transformed and divided by the interquartile range (IQR) to standardize the dynamic range of the four biomarkers. Using multiple linear regression, we examined the associations between plasma levels of pneumoproteins and four pulmonary function outcomes: post-bronchodilator FEV1 as a percentage of the predicted reference value (FEV1%predicted); post-bronchodilator FVC as a percentage of the predicted reference value (FVC%predicted); post-bronchodilator FEV1/FVC ratio; and DLco as a percentage of the predicted value (DLco%predicted) adjusted for hemoglobin and carboxyhemoglobin.

In addition to history of ever cigarette smoking (defined as having smoked at least 100 cigarettes in a lifetime), covariates included age, pack-years of cigarette smoking, BMI, CD4 count, suppressed or detectable HIV viral load, ART adherence in past week, and history of ever IDU, bacterial pneumonia, or PCP. Suppressed viral load status was defined as undetectable viral load <40 copies/mL (Abbott Molecular, Abbott Park, IL, USA). COPD was defined as a post-bronchodilator FEV_1_/FVC <0.70,[25] and abnormal DLco%predicted was defined as <80% and then divided into ≥60% but <80% (mild impairment) or <60% (moderate to severe impairment).[41] Age, BMI, and CD4 count were analyzed as continuous variables; all other predictor variables were dichotomized. Dichotomized independent variables included post-bronchodilator FEV_1_/FVC ratio (<0.70 or ≥0.70) and DLco%predicted (<60% or ≥60%). Continuous independent variables included FEV_1_%predicted, FVC%predicted, and CAT score. Age and sex were not included as predictor variables in multivariate analyses for FEV_1_%predicted, FVC%predicted, or DLco%predicted because the reference equations adjust for age and sex.

Spearman rank-order correlation coefficient was used to test initial unadjusted associations between plasma biomarkers and pulmonary function outcomes. Backward elimination was performed to determine which covariates to include in multivariable analysis for each pulmonary function outcome with pneumoprotein measurements omitted. Covariates with a significance of at least *P* = 0.05 were retained in multivariable models. Regardless of significance, history of ever cigarette smoking was included in each multivariable model because cigarette smoking is strongly associated with obstructive lung disease.[42] Once covariates were determined, separate multivariable models for each pneumoprotein were run for each pulmonary function outcome.

The two-sample t-test was used to determine whether clinical, pneumoprotein, and pulmonary function measurements differed by ART use (ART use in past week versus no ART use in past week), CD4 count (<200 cells/μL versus CD4 count ≥200 cells/μL), or HIV viral load (suppressed versus detectable) indicating extent of immune dysregulation. Pearson correlation coefficients for pairs of the different biomarkers were also calculated.

## Results

### Baseline characteristics

Overall, 65 participants were enrolled; 78.5% were male, and the median age was 51 years (**Table 1**). Of the participants, 76.9% were ever-smokers for whom the median pack-years of smoking was 23 years and 39.7% reported ever IDU (*n* = 63). Over 92% reported adherence to their ART regimen in the past week; 50.8% had an undetectable viral load, and another 29.2% had a detectable HIV viral load that was <40 copies/mL. The median CD4 cell count was 455 cells/μL, and 26.2% had a CD4 cell count <200 cells/μL. The majority (75.4%) had a history of bacterial pneumonia, and 38.5% had a history of PCP.

**Table 1:** Baseline clinical characteristics, pulmonary function tests, and pneumoprotein levels.

Baseline characteristics (*n* = 65 unless noted)
**Characteristic**	**Value**
Male sex (assigned at birth) (%)	51 (78.5%)
Median age (years) (IQR)	51 years (45–59 years)
Race/ethnicity^1^	
- White (%)	25 (38.5%)
- Black (%)	27 (41.5%)
- Hispanic (%)	10 (15.4%)
- Native Hawaiian or Other Pacific Islander (%)	6 (9.2%)
- American Indian/Alaska Native	7 (10.8%)
- Mixed (%)	5 (7.7%)
- Other (%)	6 (9.2%)
BMI, median (IQR)	26.5 (7.14)
Ever-cigarette smokers (%)	50 (76.9%)
- Median pack-years smoking, *n* = 46 (IQR)	23 years (10–44 years)
Ever injection drug use (%), *n* = 63	25 (39.7%)
Current ART use in the past week (%)	60 (92.3%)
Median CD4 count (cells/μL) (IQR)	455 cells/μL (189–651 cells/μL)
- Number of participants with CD4 count < 200 cells/μL	17 (26.2%)
Undetectable viral load (%)	33 (50.8%)
- Participants with detectable viral load < 40 copies/mL	19 (29.2%)
- Participants with detectable viral load ≥ 40 copies/mL	13 (20%)
Past history of bacterial pneumonia ever (%)	49 (75.4%)
- Past history of bacterial pneumonia in last 6 months (% out of 49 participants with past history of bacterial pneumonia)	12 (24.5%)
Past history of *Pneumocystis jirovecii* pneumonia (%)	25 (38.5%)
**Pulmonary function tests and CAT score**	**Value**
Mean post-BD FEV1%predicted (standard error)	83% (2.7%)
Mean post-BD FVC%predicted (standard error)	92% (2.2%)
Mean post-BD FEV1/FVC ratio (standard error)	0.71 (0.02)
- Number of participants with post-BD FEV1/FVC <0.70	23 (35.4%)
Mean DLco%predicted (standard error)	61% (1.8%)
- Number of participants with DLco%predicted <80%	59 (90.8%)
- Number of participants with DLco%predicted <60%	31 (47.7%)
Median CAT score, *n* = 60 (IQR)	13.5 (4.5–20.5)
**Pneumoprotein level**	**Value**
Median CC-16 level (IQR) for *n* = 60	33.9 ng/mL (21.5–46.7 ng/mL)
Median PARC/CCL-18 level (IQR)	55.0 ng/mL (38.8–86.8 ng/mL)
Median sRAGE level (IQR)	0.033 ng/mL (0.017–0.049 ng/mL)
Median SP-D level (IQR)	33 ng/mL (17–49 ng/mL)

^1^Race/ethnicity percentages do not add up to 100 because multiple choices were possible.

Abbreviations: ART = antiretroviral therapy; post-BD = post-bronchodilator; BMI = body mass index; CAT = COPD Assessment Test; CC-16 = club cell secretory protein-16; DLco%predicted = diffusing capacity for carbon monoxide corrected as percentage of predicted reference value; FEV1%predicted = forced expiratory volume in 1 second as percentage of predicted reference value; FEV1/FVC ratio = ratio of forced expiratory volume in 1 second to forced vital capacity; FVC%predicted = forced vital capacity as percentage of predicted reference value; IQR = interquartile range; PARC/CCL-18 = pulmonary and activation-regulated chemokine; SP-D = surfactant protein-D; sRAGE = soluble receptor for advanced glycation end-products.

The mean post-bronchodilator FEV_1_/FVC ratio was within normal limits but was relatively low (0.71), and 35.4% of participants had COPD (FEV_1_/FVC <0.70). The mean post-bronchodilator FEV_1_%predicted was 83%, and the mean post-bronchodilator FVC%predicted was 92%. In contrast, the mean percent-predicted DLco (DLco%predicted) was abnormal (61%), and most participants had impaired diffusing capacity: 90.8% had mildly reduced DLco%predicted (<80%), and 47.7% had moderately to severely reduced DLco%predicted (<60%). In addition, the median CAT score was 13.5 (*n* = 60), indicating a “medium” impact of COPD on a participant’s life.

### Associations between pneumoproteins and lung function or CAT score

Using Spearman correlations, significant associations were found between PARC/CCL-18 and DLco%predicted (*R* = -0.33, *P* = 0.0072) or CAT (*R* = 0.56, *P* <0.0001) and between sRAGE and DLco%predicted (*R* = -0.26, *P* = 0.037) (**Table 2**).

**Table 2:** Unadjusted associations between plasma pneumoprotein levels and pulmonary function testing outcomes using Spearman’s correlation (unless otherwise noted, *n* = 65).

	Spearman’s ρ	*P* value
**Post-BD FEV1%predicted**		
CC-16 (*n* = 60)	-0.16	0.22
PARC/CCL-18	-0.064	0.61
sRAGE	-0.059	0.64
SP-D	-0.061	0.63
**Post-BD FVC%predicted**		
CC-16 (*n* = 60)	-0.25	0.055§
PARC/CCL-18	-0.16	0.20
sRAGE	-0.066	0.60
SP-D	-0.10	0.41
**Post-BD FEV1/FVC**		
CC-16 (*n* = 60)	-0.030	0.82
PARC/CCL-18	-0.0012	0.99
sRAGE	0.047	0.71
SP-D	-0.042	0.74
**DLco%predicted**		
CC-16 (*n* = 60)	-0.17	0.18
PARC/CCL-18	-0.33	0.0072*
sRAGE	-0.26	0.037*
SP-D	0.018	0.89
**CAT**		
CC-16 (*n* = 60)	0.099	0.45
PARC/CCL-18 (*n* = 60)	0.56	<0.0001*
sRAGE (*n* = 60)	0.045	0.73
SP-D (*n* = 60)	-0.037	0.78

Note that all pneumoproteins measurements were log-transformed and divided by interquartile range. All spirometry measurements are post-bronchodilator values.

* *P* < 0.05

§ *P* ≤ 0.10

Abbreviations: post-BD = post-bronchodilator; BMI = body mass index; CAT = COPD Assessment Test; CC-16 = club cell secretory protein-16; DLco%predicted = diffusing capacity for carbon monoxide corrected as percentage of predicted reference value; FEV1%predicted = forced expiratory volume in 1 second as percentage of predicted reference value; FEV1/FVC ratio = ratio of forced expiratory volume in 1 second to forced vital capacity; FVC%predicted = forced vital capacity as percentage of predicted reference value; PARC/CCL-18 = pulmonary and activation-regulated chemokine; SP-D = surfactant protein-D; sRAGE = soluble receptor for advanced glycation end-products.

#### Spirometry

No statistically significant associations were found between any pneumoprotein and post-bronchodilator FEV_1_/FVC ratio in analyses adjusted for age, history of ever smoking, and BMI (**Table 3**). However, higher levels of CC-16 were independently associated with lower FVC%predicted (*n* = 60; *β* = -6.6, *P* = 0.012) adjusted for history of ever smoking and BMI. There was also a trend toward statistical significance between higher PARC/CCL-18 levels and lower FVC%predicted (*β* = -4.4, *P* = 0.10). Similarly, after adjusting for history of ever smoking, there were trends toward statistical significance between higher CC-16 levels and lower FEV_1_%predicted (*n* = 60; *β* = -5.8, *P* = 0.07) and higher PARC/CCL-18 levels and lower FEV_1_%predicted (*β* = -5.4, *P* = 0.10).

**Table 3:** Adjusted associations between plasma pneumoprotein levels and pulmonary function testing outcomes (unless otherwise noted, *n* = 65).

	β^❖^	*P* value	Lower 95% confidence interval	Upper 95% confidence interval
**Post-BD FEV1%predicted**^**a**^				
CC-16 (*n* = 60)	-5.8	0.07§	-12	0.49
PARC/CCL-18	-5.4	0.10§	-12	1.1
sRAGE	-2.2	0.46	-8.2	3.7
SP-D	-1.3	0.67	-7.3	4.7
**Post-BD FVC%predicted**^**b**^				
CC-16 (*n* = 60)	-6.6	0.012*	-12	-1.5
PARC/CCL-18	-4.4	0.10§	-9.8	0.91
sRAGE	-1.3	0.58	-6.2	3.5
SP-D	-1.2	0.63	-6.1	3.7
**Post-BD FEV1/FVC**^**c**^				
CC-16 (*n* = 60)	-0.0016	0.94	-0.043	0.040
PARC/CCL-18	-0.0017	0.94	-0.047	0.043
sRAGE	0.011	0.63	-0.033	0.055
SP-D	0.0013	0.95	-0.038	0.041
**DLco%predicted**^**b**^				
CC-16 (*n* = 60)	-4.5	0.044*	-8.8	-0.12
PARC/CCL-18	-8.3	<0.001*	-12	-4.2
sRAGE	-4.2	0.038*	-8.2	-0.24
SP-D	0.25	0.91	-3.9	4.4
**DLco%predicted**^**d**^				
CC-16 (*n* = 59)	-4.7	0.029*	-8.8	-0.49
PARC/CCL-18 (*n* = 64)	-6.5	0.004*	-11	-2.2
sRAGE (*n* = 64)	-1.8	0.39	-6.0	2.4
SP-D (*n* = 64)	0.82	0.69	-3.2	4.8
**DLco%predicted**^**e**^				
CC-16 (*n* = 59)	-4.5	0.045*	-9.0	-0.11
PARC/CCL-18 (*n* = 63)	-8.6	<0.001*	-13	-4.3
sRAGE (*n* = 63)	-4.5	0.053§	-9.1	0.053
SP-D (*n* = 63)	0.23	0.92	-4.1	4.5
**CAT**^**f**^				
CC-16 (*n* = 60)	1.6	0.27	-1.3	4.5
PARC/CCL-18 (*n* = 60)	6.7	<0.001*	3.9	9.5
sRAGE (*n* = 60)	-0.22	0.88	-3.1	2.6
SP-D (*n* = 60)	-1.4	0.32	-4.2	1.4

Note that all pneumoproteins measurements were log-transformed and divided by interquartile range. All spirometry measurements are post-bronchodilator values.

Variables with *P* < 0.20 in unadjusted analyses were included in multivariate analyses and retained to maximize adjusted R-squared. Regardless of significance of association, ever smoker variable was included in each multivariate model.

^a^ Adjusted for history of ever cigarette smoking

^b^ Adjusted for history of ever cigarette smoking and BMI

^c^ Adjusted for history of ever cigarette smoking, age, and BMI

^d^ Adjusted for pack-years of cigarette smoking and ART adherence in past week

^e^ Adjusted for history of ever cigarette smoking, BMI, and history of ever injection drug use

^f^ Adjusted for history of ever bacterial pneumonia

* P < 0.05

§ P ≤ 0.10

^❖^ Per IQR increase in log10 values

Abbreviations: post-BD = post-bronchodilator; BMI = body mass index; CAT = COPD Assessment Test; CC-16 = club cell secretory protein-16; DLco%predicted = diffusing capacity for carbon monoxide corrected as percentage of predicted reference value; FEV1%predicted = forced expiratory volume in 1 second as percentage of predicted reference value; FEV1/FVC ratio = ratio of forced expiratory volume in 1 second to forced vital capacity; FVC%predicted = forced vital capacity as percentage of predicted reference value; PARC/CCL-18 = pulmonary and activation-regulated chemokine; SP-D = surfactant protein-D; sRAGE = soluble receptor for advanced glycation end-products.

#### Diffusing capacity

Several pneumoproteins were independently associated with DLco%predicted adjusted for a history of ever smoking and BMI (**Table 3**). Higher PARC/CCL-18 (*β* = -8.3, *P* < 0.001), higher CC-16 (*n* = 60; *β* = -4.5, *P* = 0.044), and higher sRAGE (*β* = -4.2, *P* = 0.038) levels were all independently associated with lower DLco%predicted, adjusted for these covariates.

When a history of ever smoking was substituted with pack-years of smoking, the associations between PARC/CCL-18 and DLco%predicted (*n* = 64; *β* = -6.5, *P =* 0.004) and between CC-16 and DLco%predicted (*n* = 59; *β* = -4.7, *P* = 0.029) adjusted also for ART adherence in the past week remained statistically significant (**Table 3**). However, the adjusted association between sRAGE and DLco%predicted was no longer significant (*n* = 64; *β* = -1.8, *P* = 0.39).

Since reductions in diffusing capacity are non-specific and may be related to conditions including pulmonary vascular disease that develops as a result of injection drug use (39.7% reported ever injection drug use, *n* = 63), we performed additional analyses adjusting for IDU. When past history of IDU was forced into the multivariable models, PARC/CCL-18 (*n* = 63; *β* = -8.6, *P* < 0.001) and CC-16 (*n* = 59; *β* = -4.5, *P* = 0.045) were still significantly associated with DLco%predicted, and the association between sRAGE and DLco%predicted approached statistical significance (*n* = 63; *β* = -4.5, *P* = 0.053) adjusted also for history of ever smoking and BMI (**Table 3**).

#### CAT score

Only higher PARC/CCL-18 was independently associated with higher total CAT score (*n* = 60; *β* = 6.7, *P* < 0.001) adjusted for ever history of bacterial pneumonia (**Table 3**).

### Pneumoprotein levels and pulmonary function stratified by HIV viral suppression status, CD4 cell count, or ART use in last week

To assess whether severity of immune dysfunction in HIV infection affects pneumoprotein levels or pulmonary function levels, the data were stratified by each HIV-associated covariate: HIV viral load, CD4 cell count, or ART status. Clinical, pneumoprotein, and pulmonary function measurements did not differ significantly by these covariates except for expected associations—i.e., participants not on ART in the past week had higher viral loads (*P* = 0.0064) and a trend toward lower CD4 cell counts (*P* = 0.09).

### Correlations between biomarkers

As combinations of biomarkers have been found to be more highly correlated with inflammation or COPD clinical outcomes than single biomarkers,[43,44] correlations between biomarkers were analyzed. CC-16 was moderately correlated with PARC/CCL-18 (*R* = 0.32, *P* = 0.012), and no other correlation between biomarkers was observed.

## Discussion

In this cross-sectional exploratory analysis, we assessed the associations between plasma levels of four pneumoproteins and pulmonary function outcomes or CAT score. To our knowledge, this is the first study to characterize these associations specifically in an HIV-infected cohort. We found that higher PARC/CCL-18 levels and higher CC-16 levels were significantly associated with lower DLco%predicted. In one analysis, higher sRAGE levels were significantly associated with lower DLco%predicted. In addition, higher CC-16 levels were significantly associated with lower FVC%predicted, and higher PARC/CCL-18 levels were significantly associated with higher CAT score.

Our results support prior findings that an impaired diffusing capacity is one of the most common HIV-associated pulmonary function abnormalities.[4,9,14,45] In these studies, a moderately to severely reduced DLco was reported in 29% to 36.5% of HIV-infected participants. In our cohort, nearly 48% had a DLco%predicted <60%, a cut-off indicative of moderate (or worse) impairment in diffusing capacity. This high proportion likely is due to our recruitment strategy to enroll HIV-infected participants with acute pneumonia (but perform PFTs only after recovery from pneumonia) as a past history of bacterial pneumonia or PCP have both been associated with permanent reductions in DLco in a large multicenter study.^44^ Our results also underscore the need to understand mechanisms underlying reductions in diffusing capacity as a DLco%predicted <60% has been associated with an increased mortality in a recent multicenter study.[45]

We found that higher PARC/CCL-18 levels were significantly associated with lower DLco%predicted. While the biological role of PARC/CCL-18 is not fully understood, it is secreted primarily by innate immune cells like monocytes/macrophages and dendritic cells and is chemotactic for T-cells.[46,47] PARC/CCL-18 increases fibrosis in vivo,[48] and serum levels are elevated in idiopathic pulmonary fibrosis.[49] Higher serum PARC/CCL-18 levels have been associated not only with COPD[35] but also with increased risk for hospitalized COPD exacerbations[50] and lower DLco, higher COPD exacerbation rate, and higher BODE index (a clinical index incorporating BMI, level of airflow obstruction, dyspnea, and 6 minute walk distance)[51] in a panel with other proteins in subjects with COPD versus subjects without COPD.[44] As HIV is known to enter airway epithelial cells and increase release of inflammatory mediators,[52] our findings suggest that increased PARC/CCL-18 levels could reflect local pulmonary inflammation leading to destruction of alveolar walls and irreversible enlargement of airspaces in emphysema.

While previous studies have shown that lower CC-16 levels are associated with COPD and decreased lung function,[33,53,54] we found that higher CC-16 levels were significantly associated with lower DLco%predicted. This may indicate that the pathogenesis of HIV-associated COPD may differ from that of COPD in those without HIV due to HIV-specific inflammatory changes that impair diffusing capacity. Similar to how HIV increases chronic systemic inflammation and causes increased gut epithelial permeability and microbial translocation into the blood,[55,56] HIV might also damage the alveolar-capillary barrier, allowing more CC-16 to move into the peripheral blood. Another possible explanation is that the negative association between CC-16 and DLco may suggest the presence of interstitial lung disease as the etiology underlying the diffusing capacity abnormality. While past studies have shown an association between lower CC-16 levels and COPD, other studies have shown an association between (transient) increases in CC-16 and acute lung injuries like fire or cigarette smoke,[53,57] ozone,[58] and even certain interstitial lung diseases, idiopathic pulmonary fibrosis, and systemic sclerosis-associated interstitial lung disease.[54,59,60] CC-16 is thought to help reduce airway inflammation[61] and protect the respiratory tract from oxidative stress.[62] Larger longitudinal studies will be required to understand how CC-16 may be associated with DLco%predicted and COPD in HIV-infected populations.

Previous studies have shown that lower sRAGE levels are associated with COPD.[31,63,64] However, we found that higher sRAGE levels were associated with lower DLco%predicted in a single analysis. sRAGE is a decoy receptor of RAGE that detects stresses like hypoxia and oxidative stress and binds ligands associated with damage-associated molecular patterns to protect against inflammation.[31,63] Increasing evidence suggests that lower sRAGE levels may play a mechanistic role in worse lung function outcomes including COPD status as well as reduced DLco[31] and greater emphysema severity;[64] thus, lower sRAGE levels may reflect damage to airways or lung parenchyma respectively. Our findings may be attributed to different explanations similar to those for CC-16: HIV-specific inflammatory changes may impair diffusing capacity but also damage the alveolar-capillary barrier and allow more sRAGE to move into the peripheral blood.

Our finding that none of the pneumoproteins was significantly associated with post-bronchodilator FEV1/FVC as hypothesized may not be entirely surprising. COPD is a heterogeneous and complex disease with variable clinical presentations driven by different underlying pathogenic mechanisms.[65,66]

While these pneumoproteins have been studied in HIV-uninfected individuals, they may not reflect the specific inflammatory mediators that contribute to COPD in HIV-infected individuals. We found that DLco and spirometry measurements did not differ significantly by viral load, CD4 cell count, or ART use. Pneumoproteins associated with changes in the lung microbiome[67] or cellular senescence of T lymphocytes[9,68] could be further explored as potential biomarkers for progression or development of HIV-associated COPD.

Besides structural abnormalities like impaired DLco or decreased FEV1/FVC, functional abnormalities are common in HIV-infected individuals.[69] COPD may have highly variable clinical manifestations among different patients;[70] thus, other measurements like self-assessment of health status have become increasingly important. The CAT is a 40-point questionnaire that asks patients to self-report how COPD affects their health status.[36] While there is no “target” total CAT score, a change in 2 or more points may represent a clinically significant change in health status[71] and can help inform clinical management of COPD.[72] The association between higher PARC/CCL-18 levels and lower DLco%predicted or higher total CAT score suggests that PARC/CCL-18 may be associated with not only structural but also functional impairments in COPD, which may be an important consideration for a COPD biomarker candidate. Alternatively, HIV-infected individuals may be at higher risk for emphysema compared to other COPD subtypes.

Important limitations of our study include its cross-sectional design: without longitudinal follow-up, we could not examine causality and related COPD outcomes such as rate of FEV1 decline, COPD exacerbations, hospitalizations and mortality. While previous cohort studies did show that these pneumoproteins were associated with COPD or related outcomes during follow-up, the changes in these pneumoproteins could be due to other mechanisms besides HIV infection driving pulmonary function impairment. Also, with a relatively small sample size, the study lacked power to detect effect sizes previously reported.[33–35] Furthermore, we were unable to account for other diseases (i.e., inflammatory co-morbidities) or immunomodulatory medications that may have affected plasma pneumoprotein levels and concurrent restrictive lung disease that may have affected FVC%predicted. Future studies could examine these factors and whether these pneumoproteins are not only associated with but also causally linked with structural and functional pulmonary abnormalities in HIV-infected individuals; this would help elucidate whether statistically significant differences in these pneumoproteins lead to clinically meaningfully differences in COPD outcomes.

Our study also had several strengths. First, this is a well-characterized cohort with paired blood-pulmonary function data. Although an exploratory analysis, our study includes the major HIV-associated and COPD-associated clinical or demographic covariates identified as important predictors. Second, this is also the first study to characterize associations between pneumoproteins, specifically those that are markers for lung inflammation and injury associated with COPD, with pulmonary function measurements in an HIV-infected cohort. Our study is a preliminary step toward finding early biomarkers of adverse respiratory outcomes that could help prevent and manage COPD in high-risk populations. Finally, further insights about HIV-associated COPD may help improve understanding of both COPD and other HIV-associated comorbidities.

## Supporting information

S1 File Data including baseline clinical characteristics, pulmonary function data, and pneumoprotein levels. (CSV)
